# 한국 신규간호사가 인지하는 프리셉터의 공감과 간호실무준비도가 현장적응에 미치는 영향요인: 횡단적 서술적 조사연구

**DOI:** 10.7586/jkbns.25.004

**Published:** 2025-02-24

**Authors:** Kyeungyeun Jang, Hanna Choi

**Affiliations:** 1 Gwangju Veterans Hospital, Gwangju, Korea; 1 광주보훈병원 간호교육팀; 2 Department of Nursing Science, Nambu University, Gwangju, Korea; 2 남부대학교 간호학과

**Keywords:** Nurses, Nursing, practical, Empathy, Preceptorship, 간호사, 간호실무, 공감, 프리셉터십

## Abstract

**Purpose:**

This descriptive survey-based study was conducted to identify factors affecting new nurses’ field adaptation in relation to preceptors’ empathy and nursing practice readiness.

**Methods:**

The participants of this study were 170 new nurses who voluntarily agreed to take part in this study and had been working for 12 months or less after completing their preceptorship at three general hospitals. Data were collected using a structured questionnaire. The collected data were analyzed with the t-test, analysis of variation, the Scheffé test, Pearson correlation coefficients, and multiple regression analysis.

**Results:**

The factors that most strongly influenced field adaptation were nursing practice readiness (β = .41), above average satisfaction with the nursing job (β = .27), and below average satisfaction with the nursing job (β = −.26), in descending order. The total explanatory power of these variables was 52%.

**Conclusion:**

The findings of this study suggest that increasing new nurses’ field adaptability requires the development and application of training programs focusing on nursing knowledge, interpersonal skills, and professionalism to help them improve nursing practice readiness. In addition, policies and systems need to be developed that can improve their satisfaction with the nursing job.

## 서론

### 1. 연구의 필요성

우리나라 의료기관의 간호사 인력은 The Organization for Economic Co-operation and Development 평균의 약 53.8% 수준으로 매우 낮으며, 간호사 인력 부족 문제는 보건의료 분야에서 중요한 과제로 대두되고 있다[1]. 특히, 신규간호사의 높은 이직률(47.7%)[2]은 간호 조직의 인력관리와 간호의 질에 부정적인 영향을 미치므로, 신규간호사가 임상 환경에 잘 적응하도록 지원하는 것이 필수적이다[3]. 이렇게 신규간호사의 이직률이 높은 원인으로 간호대학에서 학습한 내용과 실제 임상현장에서의 간호 요구도가 상이해 실무현장에서 높은 기대와 부족한 역량 간의 차이로 인한 갈등 경험이 주원인이라고 하였다[4]. 이는 신규간호사의 자신감 결여, 성취감 부족, 그리고 간호현장 적응의 어려움으로 이어지고 있다[5]. 이러한 문제를 해결하기 위해서는 간호업무를 수행하기에 앞서 개인적으로는 간호 업무에 대한 준비가 철저히 이루어져야 하고, 조직차원에서는 신규간호사가 병원에 입사하여 가장 먼저, 가장 큰 도움을 받는 프리셉터의 역할이 중요하다.

병원 조직 내에서 프리셉터는 신규간호사에게 역할모델, 사회화 촉진자, 교육자로서의 역할을 수행하며[6], 이를 위해 비판적 사고능력, 의사소통 능력[7], 협력적 관계, 그리고 올바른 실무 역량을 갖추고 있어야 한다[8]. 특히 신규간호사들이 직면하는 초기의 어려움을 극복하고 성공적으로 현장에 적응하도록 돕기 위해서는 프리셉터가 신규간호사를 이해하고 배려하는 공감의 태도를 갖는 것이 필수적이다[9]. 프리셉터십의 성공적인 구현을 위해서는 효과적인 의사소통 기술이 무엇보다 중요하다[7]. 이 중에서도 공감은 긍정적인 의사소통을 이끄는 핵심 요소로, 신규간호사를 이해하고 배려하는 태도가 요구된다[9,10]. 따라서 신규간호사와 프리셉터 사이의 공감 형성은 신규간호사의 병원 적응에 있어 큰 비중을 차지한다.

신규간호사들은 대학에서의 이론 및 실습 교육과 의료기관에서의 실무 교육을 통해 임상 현장에서 간호사로서 업무를 수행하는 데 필요한 기술과 지식을 습득한다. 또한 전문직 정체성, 협동 능력, 인간관계 형성 능력을 함양하여 임상에서 독립적으로 업무를 수행할 준비를 한다[11]. 하지만 신규간호사의 간호 실무에 대한 준비가 부족한 경우, 의료 사고 발생 증가와 환자 회복 지연 등 환자 안전에 부정적인 영향을 미칠 수 있다[12]. 따라서 신규간호사가 임상 현장에서 스스로 간호를 수행하기 시작하는 시점에 이들의 실무 준비 정도를 점검하는 것이 필요하다. 또한 병원은 간호사가 임상에서 부족한 부분을 보완하기 위해 추가 교육과 관리체계를 마련하여 신규간호사가 성공적으로 임상에 적응할 수 있도록 지원해야 한다[13].

현장 적응은 조직 구성원이 맡은 역할 수행에 필요한 가치관, 기술, 그리고 조직 내 사회적 지식을 학습하는 과정이다[14]. 신규간호사의 효과적인 조직사회화는 개인의 태도와 행동을 변화시켜 현장 적응력을 높이고 이직을 예방하는 데 중요한 역할을 하고 있다[15]. 특히 입사 후 1년은 신규간호사의 학습, 태도, 그리고 생산성에 영향을 미치는 중요한 기간으로, 병원 환경과 임상 현장에 잘 적응할 수 있도록 신규간호사를 이해하고 지원하는 것이 필요하다[16]. 따라서 병원 내에서 프리셉터의 역할은 특히 중요하다. 프리셉터는 신규간호사가 현장에서 요구되는 기술과 지식을 습득할 수 있도록 도와주며, 그들의 감정적, 사회적 적응을 지원하는 중요한 역할을 한다. 프리셉터와의 긍정적인 관계와 공감은 신규간호사가 현장에 잘 적응하도록 돕고, 이직 의도를 감소시킬 수 있다. 이와 같은 지원은 신규간호사의 자신감을 증진시키고, 궁극적으로 환자에게 질 높은 간호 제공에도 기여하게 된다.

지금까지 신규간호사의 현장적응 관련 선행연구에서는 직장 내 괴롭힘 경험, 긍정심리자본[17], 임상수행능력, 전문직업성[18], 자아존중감, 회복탄력성[19] 등의 개인이 경험하거나 인지하는 다양한 개별 변수들과 현장적응에 대한 연구가 부분적으로 다뤄져 왔다. 그러나 개별 변수 중심의 연구 외에도 신규간호사의 현장적응이 기초간호학 교육과 조직 차원의 지원이 어떻게 연계될 수 있는지에 대한 연구는 부족하다. 또한, 신규간호사들이 임상현장에서 기초간호학 교육이 필요할 경우 교육 내용에 맞는 효과적인 교육지원 방안과 영향 요인 탐색이 요구된다. 따라서, 선행연구를 바탕으로 현장적응에 관계가 있었던 변수들을 종합적으로 규명할 필요가 있다. 이에 본 연구는 신규간호사가 인지한 프리셉터의 공감과 간호실무준비도가 현장 적응에 미치는 영향을 파악하고자 한다. 이를 바탕으로 신규간호사들이 임상현장에서 기초간호학적 지식을 쉽게 활용할 수 있도록 교육 설계 시 임상적용을 고려한 교육 전략을 마련하는데 근거를 제공하고, 지식 습득을 넘어 실무 적용 능력을 강화하는 방향으로 교육 개선에 기여하고자 한다.

### 2. 연구의 목적

본 연구의 목적은 신규간호사가 인지하는 프리셉터의 공감과 간호실무준비도가 현장적응에 미치는 영향을 파악하고자 함이다. 구체적인 목적은 다음과 같다.

첫째, 신규간호사가 인지하는 프리셉터의 공감, 간호실무준비도 및 현장적응의 정도를 파악한다.

둘째, 일반적 특성에 따른 현장적응의 차이를 파악한다.

셋째, 신규간호사가 인지하는 프리셉터의 공감, 간호실무준비도 및 현장적응 간의 상관관계를 파악한다.

넷째, 신규간호사의 현장적응에 영향을 미치는 유의한 영향요인을 확인한다.

## 연구 방법

### 1. 연구 설계

본 연구는 신규간호사가 인지하는 프리셉터의 공감과 신규간호사의 간호실무준비도가 현장적응에 미치는 영향을 파악하기 위해 수행된 횡단적 서술적 조사연구이다.

### 2. 연구 대상

본 연구의 대상자는 3개 광역시의 프리셉터십을 운영 중인 500병상 이상 종합병원에서 정규직 간호사로, 프리셉터와 1:1 교육을 받은 후 근무한 지 12개월 이하이며, 연구 목적을 이해하고 연구 참여에 동의한 자를 포함하였다. 프리셉터십 교육과정을 마치지 않은 간호사는 제외하였다. 연구대상자 수는 G*Power program 3.1.9.7 [20]을 이용하여 산출하였다. 선행연구[7]에 근거하여 상관관계 및 회귀분석을 위한 중간 효과크기(d)= .15, 검정력(1-β)은 .90, 유의수준 α = .05, 예측변수 13개(신규간호사가 인지하는 프리셉터의 공감, 간호실무준비도, 일반적 특성 11개)를 포함했을 때 필요한 최소 표본 수는 162명으로 계산되었다. 약 10%의 탈락률을 고려하여 총 180부의 설문지를 배포하였으며, 이 중 177부가 회수되었다. 응답이 불성실한 7부를 제외한 170부를 최종 분석에 사용하였다.

### 3. 연구 도구

#### 1) 신규간호사가 인지하는 프리셉터의 공감

신규간호사가 인지하는 프리셉터의 공감은 Kim과 Cho [21]이 개발한 타인평정 공감척도를 사용하여 측정하였다. 이 도구는 공감의 시작(4문항), 공감의 과정(4문항), 공감의 결과(3문항)의 3개 영역으로 구성된 총 11문항의 자기보고식 설문 도구이다. 각 문항은 Likert 5점 척도로 ‘전혀 그렇지 않다’ 0점에서 ‘매우 그렇다’ 4점까지 평가하며, 총 점수 범위는 최소 0점에서 최대 44점이다. 점수가 높을수록 공감의 정도가 높은 것을 의미한다. Kim과 Cho [21]의 연구에서 도구의 신뢰도는 Cronbach's α = .86으로 나타났으며, 본 연구에서는 Cronbach's α = .93이었다.

#### 2) 간호실무준비도

간호실무준비도는 Kim과 Shin [13]이 개발한 도구를 사용하여 측정하였다. 이 도구는 임상판단과 간호수행(16문항), 전문적 가치와 태도(8문항), 환자 중심적 태도(5문항), 자기조절(3문항), 협력적 대인관계(3문항)의 5개 영역으로 구성된 총 35문항으로 이루어져 있다. 각 문항은 Likert 4점 척도로 ‘전혀 아니다’ 1점에서 ‘매우 그렇다’ 4점까지 평가하며, 총 점수 범위는 최소 35점에서 최대 140점이다. 점수가 높을수록 간호실무준비도가 높은 것을 의미한다. Kim과 Shin [13]의 연구에서 도구의 신뢰도는 Cronbach's α = .89로 나타났으며, 본 연구에서는 Cronbach's α = .92이었다.

#### 3) 현장적응

현장적응은 Sohn et al. [14]이 개발한 도구를 사용하여 측정하였다. 이 도구는 개인적 특성(8문항), 단체적 특성(8문항), 직업정체성(3문항), 직무수행(5문항), 직무만족(5문항), 조직몰입(5문항), 소진(5문항)의 7개 영역으로 구성된 총 39문항으로 이루어져 있다. 각 문항은 Likert 5점 척도로 ‘전혀 그렇지 않다’ 1점에서 ‘매우 그렇다’ 5점까지 평가하며, 총 점수 범위는 최소 39점에서 최대 195점이다. 점수가 높을수록 현장적응 정도가 높은 것을 의미한다. Sohn et al. [14]의 연구에서 도구의 신뢰도는 Cronbach's α = .98로 나타났으며, 본 연구에서는 Cronbach's α = .88이었다.

### 4. 자료 수집

본 연구의 자료수집은 남부대학교 생명윤리위원회의 승인을 받은 후 2021년 7월 20일부터 2021년 8월 13일까지 진행되었다. 자료수집 대상은 중앙보훈병원, 부산보훈병원, 광주보훈병원에 근무하는 간호사였다. 연구자는 각 병원의 협조를 얻은 후 연구 목적, 방법, 내용을 간호사들에게 설명하였으며, 연구 참여를 원하는 간호사를 대상으로 연구모집 공고문을 배포하였다. 자발적으로 연구 참여 의사를 밝힌 간호사에게 연구보조자가 연구 참여 동의서를 받았다. 자료수집 절차로, 연구 참여자는 연구보조자로부터 설문지 작성 방법에 대한 설명을 들은 후 설문지를 작성하였다. 작성 완료된 설문지는 불투명 봉투에 넣어 밀봉하도록 하였으며, 연구자는 직접 병원을 방문하거나 우편을 통해 설문지를 회수하였다. 회수된 설문지는 연구자가 즉시 확인하여 응답이 불성실한 설문지는 제외하고, 최종적으로 170부의 설문지를 분석에 사용하였다.

### 5. 자료 분석

수집된 자료는 SPSS version 26.0 (IBM Co., Armonk, NY, USA)을 이용하여 분석하였다. 대상자의 일반적 특성과 주요 변수의 수준은 평균, 표준편차, 빈도와 백분율로 분석하였다. 일반적 특성에 따른 현장적응의 차이는 정규분포 가정을 만족하지 않아 비모수 검정인 Mann-Whitney U test, Kruskal Wallis test와 independent t-test, one-way ANOVA로 분석하였고, 사후분석은 Scheffé test로 하였다. 대상자가 인지하는 프리셉터의 공감, 간호실무준비도 및 현장적응 간의 상관관계는 Pearson correlation coefficients를 이용하여 분석하였다. 대상자의 현장적응에 영향을 미치는 요인을 파악하기 위해 다중회귀분석(multiple regression)을 실시하였다. 희망부서 배치 여부, 프리셉터와의 관계, 간호직 만족도는 더미변수화하여 분석에 포함하였다. 모든 분석에서 유의수준은 *p* < .05로 설정하였으며, 다중공선성 문제를 확인하기 위해 분산팽창계수(variance inflation factor, VIF)를 검토하였다. VIF 값이 10 미만으로 나타나 다중공선성의 문제는 없는 것으로 확인되었다.

### 6. 윤리적 고려

본 연구는 연구대상자의 윤리적 보호를 위하여 남부대학교 생명윤리위원회의 승인(IRB No. 104147B-2021-HR-007)과 간호부서장의 사전 허락을 받은 후 시행되었다. 대상자에게 연구 참여로 인한 불편감, 신분 비밀 보장 및 사생활 보호에 대해 안내하였으며, 연구 참여는 자발적임을 강조하고, 연구 과정 중 언제든지 중단할 수 있으며 어떠한 위험과 불이익도 발생하지 않을 것임을 설명하였다. 설문 작성 전 연구보조자가 서면 동의서를 받은 후, 동의서를 작성한 대상자만 설문을 참여할 수 있었다. 모든 설문지는 익명으로 처리되었으며, 응답 완료 후 대상자에게 소정의 답례품을 제공하였다. 수집된 자료는 개인정보 보호법에 따라 철저히 관리될 예정이며, 연구 종료 후 3년이 경과한 시점에 관련 문서는 파쇄하여 영구적으로 삭제할 예정임을 설명하였다.

## 연구 결과

### 1. 대상자의 특성

본 연구대상자는 총 170명이었으며, 성별은 여성 146명(85.9%), 연령은 21∼25세가 81명(47.7%)으로 가장 많았다. 결혼상태는 미혼이 163명(95.9%), 학력은 대학교 졸업이 157명(92.3%)으로 많았다. 현 병원 근무기간은 4∼6개월(31.8%), 근무부서는 병동이 128명(75.2%)이었으며, 프리셉터십 기간은 9∼12주가 98명(57.7%)으로 가장 많았다. 희망부서 배치 여부는 ‘예’가 100명(58.8%)으로 많았고, 프리셉터와의 관계는 만족이 114명(67.1%)이었다. 근무형태는 3교대가 161명(94.7%)을 차지했으며, 간호직 만족도는 보통이 90명(52.9%)으로 가장 많았다(Table 1).

### 2. 대상자가 인지하는 프리셉터의 공감, 간호실무준비도, 현장적응 정도

대상자가 인지하는 프리셉터의 공감은 평균 4점 만점에 2.50 ± 0.61점으로 ‘공감의 시작’ 영역이 2.58 ± 0.65점으로 가장 높았으며, ‘공감의 과정’이 2.40 ± 0.70점으로 가장 낮은 점수를 보였다. 간호실무준비도는 4점 만점에 3.00 ± 0.28점으로 ‘환자중심 태도’ 영역에서 3.32 ± 0.38점으로 가장 높았으며, ‘전문가적 가치와 태도’ 영역에서 2.81 ± 0.46점으로 가장 낮은 점수를 보였다. 현장적응은 5점 만점에 3.17 ± 0.37점으로 ‘개인적인 특성’ 영역에서 3.69 ± 0.51점으로 가장 높았으며, ‘직무수행’ 영역에서 2.47 ± 0.69점으로 가장 낮은 점수를 보였다(Table 2).

### 3. 대상자의 특성에 따른 현장적응의 차이

일반적 특성에 따른 현장적응의 차이를 분석한 결과, 희망부서 배치 여부(t = 2.04, *p* = .043), 프리셉터와의 관계(F = 8.19, *p* < .001), 간호직 만족(F = 49.51, *p* < .001)에 따라 유의한 차이가 있었다. 희망부서에 배치된 경우가 그렇지 않은 경우보다 현장적응 점수가 높았으며, 프리셉터와의 관계에 만족할 때 현장적응 점수가 높았다. 간호직 만족도는 만족하는 경우가 가장 높았고, 보통, 불만족 순으로 현장적응 점수가 높게 나타났다(Table 3).

### 4. 대상자가 인지하는 프리셉터의 공감, 간호실무준비도, 현장적응 간의 상관관계

대상자가 인지하는 프리셉터의 공감은 간호실무준비도(r = .44, *p* < .001), 현장적응(r = .45, *p* < .001)과 양의 상관관계가 있었으며, 간호실무준비도는 현장적응과 양의 상관관계가 있었다(r = .61, *p* < .001) (Table 4).

### 5. 대상자의 현장적응에 미치는 영향요인

현장적응에 영향을 미치는 요인을 규명하기 위해, 일반적 특성 중 현장적응에 유의한 관계를 보인 변수인 희망부서 배치 여부, 프리셉터와의 관계, 간호직 만족도와 신규간호사가 인지하는 프리셉터의 공감, 간호실무준비도를 포함하여 다중회귀분석을 실시하였다. 회귀모형의 적합성 검정은 잔차분석을 통해 정규성 분포와 등분산성을 검토하였다. 희망부서 배치 여부, 프리셉터와의 관계, 간호직 만족도는 더미변수화하여 분석에 포함하였다. 종속변수의 자기상관과 독립변수 간 다중공선성을 확인한 후, 회귀분석을 진행하였다. 종속변수의 Durbin-Watson 지수는 1.66로 2에 가까워 자기 상관이 없고 독립적이었다. 독립변수 간 VIF 지수는 1.15∼1.76으로 10 미만으로 다중공선성이 없었으며, 잔차분석 결과 정규성 및 등분산성이 입증되었다. 따라서 본 데이터는 회귀분석을 실시하기에 적합한 것으로 확인되었다. 다중회귀분석 결과, 간호실무준비도(β = .41)와 간호직 만족도가 보통에 비해 만족하는 경우(β = .27), 보통에 비해 불만족인 경우(β = -.26) 순으로 현장적응에 영향을 미쳤으며, 이들 변수의 설명력은 52%였다(Table 5).

## 논의

본 연구는 신규간호사가 인지하는 프리셉터의 공감, 간호실무준비도 및 현장적응 간의 관계를 파악하고, 현장적응에 미치는 요인을 확인하기 위한 황단적 서술적 조사연구이다. 신규간호사의 현장적응력을 증진시키기 위한 중재 개발의 기초자료 제공을 목표로 시행되었다.

본 연구에서 현장적응은 5점 만점에 평균 3.17점으로 나타났으며, 이는 신규간호사를 상으로 한 선행연구[17]의 2.98점과 비슷한 수준이었다. 또한, 본 연구대상자와 비슷한 규모의 병원 간호사를 대상으로 한 연구들보다 다소 낮은 결과를 보였다[22,23]. 이는 본 연구 대상자들의 대부분은 4학년 때 코로나19 팬데믹을 경험하며 임상실습의 단절 혹은 비대면 전환으로 핵심간호술 수행이나 취업준비에 어려움을 겪었기 때문으로 생각된다. 코로나19 팬데믹으로 인해 임상실습이 중단되거나 제한되면서 대학 시절 코로나19 팬데믹을 겪은 신규간호사의 병원 적응에 더 큰 어려움 겪고 있을 것으로 사료된다. 팬데믹 상황에서 임상실습 제한 등의 요인이 신규 간호사의 병원 현장적응을 방해하고 있으므로, 이러한 상황에 대비할 수 있는 대책이 필요하다[24].

본 연구에서 현장적응에 가장 큰 영향을 미친 간호실무준비도는 4점 만점에 평균 3.0점으로, 상급종합병원 신규간호사를 대상으로 한 연구에서 평균 2.9점으로 비슷한 수준을 보였다[13]. 간호실무준비도의 세부 요인에서도 ‘환자중심적 태도’와 ‘자기조절’ 요인 문항 평균이 각각 3.32점, 3.28점으로 높게 나타났으며, 그 다음이 ‘협력적 대인관계’ 3.19점, ‘임상판단과 간호수행’ 2.91점, ‘전문직 가치와 태도’가 2.81점으로 가장 낮게 나타나 상급종합병원과 차이를 보이지 않았다[25]. 신규간호사는 이론과 실습 교육을 통해 기초적인 지식과 기술을 습득하지만, 복잡한 임상업무 수행을 위해서는 임상판단, 간호수행, 협력적 대인관계 등 다양한 역량이 요구된다[11]. 이와 같이 병원의 종류와 크기에 관계없이 신규간호사는 상대적으로 기본 간호 지식과 기술이 부족하여 대처 능력이 미숙해지는 경우가 많고, 이는 간호수행에 어려움을 주고 간호 질 저하로 이어질 수 있다. 선행연구에 따르면 간호실무준비도가 높은 신규간호사는 현장적응과 직무 성취도가 높고, 반대로 준비도가 부족한 경우 핵심 업무에서 어려움을 겪고 자신감 저하로 이어졌다[13]. 또한 간호실무준비도가 높을수록 현실충격은 낮고, 현장적응이 높아진다고 보고되었다[26]. 따라서 병원에서는 신규간호사의 현장 적응력 향상과 환자안전 강화를 위하여 간호실무준비도를 측정하고, 교육대상자를 선별하여 시기별 특성에 맞는 교육프로그램을 제공하는 것이 신규간호사의 현장적응을 높이는 데 도움이 될 것이다. 신규간호사의 다양한 비대면 온라인 교육 프로그램이나 Virtual Reality, Augmented Reality 등의 최신 보건의료기술을 활용하여 원하는 시간에 실감나는 자기주도 학습이 가능한 콘텐츠 개발로 신규간호사의 간호실무준비도를 높일 필요가 있다[24].

대상자의 특성에 따른 현장적응의 차이를 분석한 결과 간호직 만족도, 희망부서 배치 여부, 프리셉터와의 관계에서 유의미한 차이가 있었다. 특히 간호직 만족도는 간호실무준비도 다음으로 현장적응에 영향을 미치는 요인으로, 만족하는 경우 현장적응 점수가 높았다. 이는 간호직에 대한 만족도가 높을수록 현장적응 수준이 높다는 결과와 일치한다[19,27]. 이러한 결과는 간호직 만족도가 업무를 긍정적으로 받아들이고 처리하는데 차이가 있는 결과라고 보았다[19]. 또한, 희망부서에 배치된 경우와 프리셉터와의 관계가 만족스러운 경우에도 현장적응 수준이 높았다. 이는 선행연구[28,29]와 일치하는 결과로, 희망부서 배치가 현장적응에 업무 환경과 역할에 대한 기대와 현실의 간극을 줄이고, 업무 동기를 높이는 방식으로 긍정적인 영향을 미친다고 볼 수 있다. 또한, 프리셉터와의 관계가 좋을수록 업무 수행에 대한 실질적인 지원과 정서적 지지를 제공받아, 자신감을 갖고 새로운 환경에 적응하는 데 도움을 주는 방식으로 현장적응력이 향상됨으로 영향을 줄 수 있을 것이다. 따라서 신규간호사의 현장적응을 높이기 위해서는 희망부서 배치, 프리셉터 관계 증진과 더불어 간호직 만족도 증진이 필요할 것이라 생각된다.

프리셉터의 공감, 간호실무준비도와 현장적응 간의 상관관계를 살펴본 결과, 프리셉터의 공감은 간호실무준비도, 현장적응과 양의 상관관계를 보였으며, 간호실무준비도는 현장적응과 양의 상관관계를 나타냈다. 이는 프리셉터의 공감과 간호실무준비도가 높을수록 현장적응이 향상된다는 것을 시사한다. 따라서 신규간호사의 현장적응을 돕기 위해 프리셉터의 공감 능력 향상 교육과 간호실무준비도를 높이는 병원 차원의 체계적인 지원이 필요하다.

본 연구에서 신규간호사가 인지하는 프리셉터의 공감은 4점 만점에 평균 2.5점으로 중간 이상의 수준이었다. 상급종합병원 신규간호사를 대상으로 한 선행연구에서, 인지된 프리셉터의 공감이 높을수록 직무만족도가 높다는 결과가 있었다[8]. 이는 프리셉터와의 관계 형성에서 공감 능력이 긍정적인 영향을 미쳐 신규간호사의 현장적응에 도움이 되었을 것이라고 생각된다. 본 연구에서 신규간호사가 인지하는 프리셉터의 공감이 높을수록 현장적응 정도가 높아짐은 확인하였으나, 현장적응에 영향을 미치는 요인은 아님을 확인하였다. 프리셉터의 공감은 간호실무준비도와 현장적응과 양의 상관관계를 보였으나, 다중회귀분석에서는 직접적인 효과가 확인되지 않았다. 현장적응은 단순히 프리셉터의 공감뿐만 아니라 조직의 환경과 같은 다양한 요인이 복합적으로 작용하거나 공감 측정 도구의 한계가 저해요인으로 작용했을 가능성도 고려된다. 또한, 프리셉터의 공감이 실제 지도와 연계되지 않아 신규간호사의 적응에 미치는 영향이 제한적일 수 있다. 따라서 조직 내 프리셉터 개인의 지지도 중요하지만 조직에서의 전반적인 지지와 격려가 필요할 것으로 보이며, 신규간호사가 인지하는 프리셉터의 공감과 현장적응 간의 종합적인 분석과 반복연구가 요구된다[30].

본 연구는 신규간호사의 현장적응에 영향을 미치는 요인으로 간호실무준비도와 간호직 만족도를 확인하였으며, 이들이 현장적응을 51.9% 설명할 수 있음을 발견하였다. 신규간호사들은 업무 미숙으로 환자에게 해를 끼치는 것을 걱정하므로, 간호실무준비도를 높이는 현장적응 프로그램이 필요하다[31]. 따라서 신규간호사의 간호실무에 대한 준비도를 평가하고, 이들의 교육 요구도와 영역별 취약점을 파악하여 지원하는 것이 신규간호사의 현장적응에 도움이 될 것으로 사료된다. 또한 신규간호사의 간호직에 대한 만족도를 증가시킬 수 있는 간호 조직의 다양한 정책적 방안 마련이 필요하다. 신규간호사를 배려한 정책적 방안을 유지 및 관리하여 신규간호사의 현장적응력을 증진시킨다면, 의료기관의 간호인력 관리에 도움이 될 것이다. 이는 임상현장에서 환자안전과 양질의 간호를 제공에도 일조할 수 있을 것이라고 사료된다.

이에 본 연구는 신규간호사의 현장적응에 영향을 미치는 요인을 밝힘으로써 현장적응력 향상을 위한 중재프로그램 개발의 학문적 근거자료를 제시하였다는 점에서 의의가 있다. 이로써 기초간호학 교육적 측면에서 본 연구는 임상 현장에서 기초간호학적 지식을 효과적으로 활용할 수 있도록 교육 설계 시 실무적용을 반영한 전략을 수립하는데 근거를 제공하였다. 이를 통해 단순한 이론 학습을 넘어, 실무에서의 활용 능력을 강화하는 방향으로 교육을 개선하는데 기여할 수 있을 것이다. 또한, 기초간호학 연구적 측면에서는 신규간호사의 실무 준비도를 평가하고 강화할 수 있는 구체적인 교육 목표 설정을 지원하며, 이를 통해 간호사 현장적응과 관련된 다양한 변수 탐구뿐만 아니라 제도 개선 및 방안 마련을 위한 기초자료로 활용될 수 있을 것이다.

본 연구결과를 토대로 다음과 같이 제언한다. 첫째, 본 연구는 3개 광역시 3곳의 종합병원 신규간호사를 대상으로 하였기 때문에 다양한 지역과 의료기관으로 대상을 확장하여 반복적으로 연구결과를 검증할 필요가 있다. 이와 함께, 간호직 만족도 등을 포함하여 프리셉터십 및 신규 간호사의 적응과 관련된 기초 연구를 강화하여 이론적 기반을 확립하고, 이를 바탕으로 임상 실무와 연계된 실증적 연구를 지속적으로 수행할 필요가 있다.

## 결론

본 연구는 신규간호사가 인지하는 프리셉터의 공감과 간호실무준비도가 현장적응에 영향을 미치는 요인을 파악하기 위한 서술적 조사연구이다. 본 연구의 결과 신규간호사의 현장적응에 영향을 미치는 요인은 간호실무준비도, 간호직 만족으로 나타났으며 간호실무준비도가 높을수록, 간호직에 대한 만족이 높을수록 신규간호사들이 현장적응을 잘하는 것을 확인하였다.

따라서 신규간호사의 현장적응력 향상을 위하여 간호실무준비도를 높일 수 있는 교육 프로그램을 개발하고, 간호직에 대한 만족도를 높일 수 있는 다양한 전략을 적용이 필요하다. 특히, 프리셉터의 공감력 강화를 통한 신규간호사와의 소통 및 신규간호사의 간호실무준비도를 상승시킨다면 의료기관의 간호인력 관리에 도움이 되어 임상현장에서 환자안전과 양질의 간호를 제공하는데 도움이 될 수 있을 것이다.

## Figures and Tables

**Table 1.** General Characteristics of Participants (N = 170)

Characteristics	Categories	n (%)	M ± SD
Sex	Men	24 (14.1)	
	Women	146 (85.9)	
Age (yr)	21 ~ 25	81 (47.7)	
	26 ~ 30	76 (44.6)	
	≥ 31	13 (7.7)	
Marital status	Single	163 (95.9)	
	Married	7 (4.1)	
Education	College (3 years)	13 (7.7)	
	University (4 years)	157 (92.3)	
Employment period (mon)	1 ~ 3	29 (17.1)	7.04 ± 3.07
	4 ~ 6	54 (31.8)	
	7 ~ 9	42 (24.6)	
	10 ~ 12	45 (26.5)	
Department	Ward	128 (75.2)	
	Intensive care unit	21 (12.4)	
	Others^†^	21 (12.4)	
Period of preceptorship (wk)	≤ 4	31 (18.2)	9.16 ± 3.03
	5 ~ 8	41 (24.1)	
	9 ~ 12	98 (57.7)	
Assignment to preferred department	Yes	100 (58.8)	
	No	70 (41.2)	
Relationship with preceptors	Dissatisfied	6 (3.5)	
	Neutral	50 (29.4)	
	Satisfied	114 (67.1)	
Shift work	Three shifts	161 (94.7)	
	Fixed shift, others^‡^	9 (5.3)	
Satisfaction with nursing	Dissatisfied	17 (10.0)	
	Neutral	90 (52.9)	
	Satisfied	63 (37.1)	

M = Mean; SD = Standard deviation.

† Operating room, recovery room, artificial kidney unit, emergency unit;

‡ Flexible working arrangement.

**Table 2.** Degree of Preceptor’s Empathy Perceived by New Nurses, Nursing Practice Readiness, and Field Adaptation (N = 170)

Variables	M ± SD	Min	Max	Range
Preceptor’s empathy perceived by new nurses	2.50 ± 0.61	1.00	3.91	0 ~ 4
Initiation of empathy	2.58 ± 0.65	0.75	4.00	
Process of empathy	2.40 ± 0.70	0.75	4.00	
Outcomes of empathy	2.52 ± 0.66	1.00	4.00	
Nursing practice readiness	3.00 ± 0.28	2.37	3.83	1 ~ 4
Patient-oriented attitude	3.32 ± 0.38	1.50	4.00	
Self-regulation	3.28 ± 0.40	2.00	4.00	
Cooperative interpersonal relationship	3.19 ± 0.38	2.00	4.00	
Clinical judgment and nursing performance	2.91 ± 0.28	2.00	3.81	
Professional values and attitudes	2.81 ± 0.46	2.80	4.00	
Field adaptation	3.17 ± 0.37	2.21	4.26	1 ~ 5
Personal characteristics	3.69 ± 0.51	1.63	4.75	
Professional identity	3.65 ± 0.68	1.00	5.00	
Organizational characteristics	3.42 ± 0.45	2.13	4.75	
Job satisfaction	3.20 ± 0.54	1.40	4.80	
Organizational commitment	2.87 ± 0.67	1.00	4.80	
Burn out	2.59 ± 0.65	1.00	4.60	
Job performance	2.47 ± 0.69	1.00	4.80	

M = Mean; SD = Standard deviation; Min = Minimum; Max = Maximum.

**Table 3.** Differences in Field Adaptation According to Participants’ Characteristics (N = 170)

Characteristics	Categories	Field adaptation		
		M ± SD	t/F(*p*)	Scheffé
Sex	Men	3.16 ± 0.42	−0.05 (.962)	
	Women	3.17 ± 0.36		
Age (yr)	21 ~ 25	3.20 ± 0.40	0.79 (.454)	
	26 ~ 30	3.14 ± 0.35		
	≥ 31	3.09 ± 0.23		
Marital status	Single	3.17 ± 0.37	0.94 (.347)	
	Married	3.04 ± 0.27		
Education	College	3.13 ± 0.27	−0.36 (.716)	
	University	3.17 ± 0.38		
Employment period (mon)	1 ~ 3	3.22 ± 0.41	0.63 (.596)	
	4 ~ 6	3.18 ± 0.36		
	7 ~ 9	3.17 ± 0.38		
	10 ~ 12	3.11 ± 0.36		
Department	Word	3.16 ± 0.38	0.16 (.856)	
	Intensive care unit	3.21 ± 0.37		
	Others^†^	3.17 ± 0.35		
Period of preceptorship (wk)	≤ 4	3.13 ± 0.37	0.20 (.823)	
	5 ~ 8	3.16 ± 0.33		
	9 ~ 12	3.18 ± 0.39		
Assignment to preferred department	Yes	3.21 ± 0.33	2.04 (.043)	
	No	3.10 ± 0.41		
Relationship with preceptors	Dissatisfied^a^	2.94 ± 0.55	8.19 (< .001)	b < c
	Neutral^b^	3.02 ± 0.32		
	Satisfied^c^	3.24 ± 0.36		
Shift work	Three shifts	3.17 ± 0.37	0.64 (.521)	
	Fixed shift, others^‡^	3.09 ± 0.47		
Satisfaction with nursing	Dissatisfied^a^	2.70 ± 0.37	49.51 (< .001)	a< b < c
	Neutral^b^	3.08 ± 0.24		
	Satisfied^c^	3.42 ± 0.34		

M = Mean; SD = Standard deviation.

† Operating room, recovery room, artificial kidney unit, emergency unit;

‡ Flexible working arrangement;

**Table 4.** Correlations among Preceptor’s Empathy Perceived by New Nurses, Nursing Practice Readiness, and Field Adaptation (N = 170)

Variables	Preceptor’s empathy perceived by new nurses	Nursing practice readiness	Field adaptation
	r (*p*)		
Nursing practice readiness	.44 (< .001)	1	-
Field adaptation	.45 (< .001)	.61 (< .001)	1
			

**Table 5.** Factors Influencing Field Adaptation (N = 170)

Variables	B	β	t	*p*	VIF
(constant)	1.35		5.72	< .001	
Assignment to preferred department (ref = No)	−0.06	−.08	−1.47	.143	1.15
Relationship with preceptors (dissatisfied) (ref = Neutral)	0.00	.00	−0.01	.989	1.20
Relationship with preceptors (satisfied) (ref = Neutral)	0.03	.04	0.57	.572	1.62
Satisfaction with nursing (dissatisfied) (ref = Neutral)	−0.32	−.26	−4.26	< .001	1.28
Satisfaction with nursing (satisfied) (ref = Neutral)	0.21	.27	4.19	< .001	1.45
Preceptor’s empathy perceived by new nurses	0.05	.08	1.09	.276	1.76
Nursing practice readiness	0.56	.41	6.64	< .001	1.37
Durbin-Watson = 1.66, R²= .54, Adj R²= .52					
F (*p)* = 27.02 (< .001)					

ref = Reference; VIF = Variance inflation factor.

## Data Availability

Please contact the corresponding author for data availability.
